# Risk factors for SARS-CoV-2 infection during the early stages of the COVID-19 pandemic: a systematic literature review

**DOI:** 10.3389/fpubh.2023.1178167

**Published:** 2023-07-31

**Authors:** Matthew Harris, John Hart, Oashe Bhattacharya, Fiona M. Russell

**Affiliations:** ^1^Melbourne School of Population and Global Health, The University of Melbourne, Parkville, VIC, Australia; ^2^Asia-Pacific Health Group, Infection, Immunity and Global Health, Murdoch Children's Research Institute, Parkville, VIC, Australia; ^3^Department of Paediatrics, The University of Melbourne, Parkville, VIC, Australia; ^4^Centre for International Child Health, Department of Paediatrics, The University of Melbourne, Parkville, VIC, Australia

**Keywords:** SARS-CoV-2, COVID-19, risk factors, infection, variant of concern (VoC)

## Abstract

**Introduction:**

Identifying SARS-CoV-2 infection risk factors allows targeted public health and social measures (PHSM). As new, more transmissible variants of concern (VoC) emerge, vaccination rates increase and PHSM are eased, it is important to understand any potential change to infection risk factors. The aim of this systematic literature review is to describe the risk factors for SARS-CoV-2 infection by VoC.

**Methods:**

A literature search was performed in MEDLINE, PubMed and Embase databases on 5 May 2022. Eligibility included: observational studies published in English after 1 January 2020; any age group; the outcome of SARS-CoV-2 infection; and any potential risk factors investigated in the study. Results were synthesized into a narrative summary with respect to measures of association, by VoC. ROBINS-E tool was utilized for risk of bias assessment.

**Results:**

Of 6,197 studies retrieved, 43 studies were included after screening. Common risk factors included older age, minority ethnic group, low socioeconomic status, male gender, increased household size, occupation/lower income level, inability to work from home, public transport use, and lower education level. Most studies were undertaken when the ancestral strain was predominant. Many studies had some selection bias due to testing criteria and limited laboratory capacity.

**Conclusion:**

Understanding who is at risk enables the development of strategies that target priority groups at each of the different stages of a pandemic and helps inform vaccination strategies and other interventions which may also inform public health responses to future respiratory infection outbreaks. While it was not possible to determine changes to infection risk by recent VoC in this review, the risk factors identified will add to the overall understanding of the groups who are at greatest risk of infection in the early stages of a respiratory virus outbreak.

**Systematic review registration:**

https://www.crd.york.ac.uk/prospero/display_record.php?ID=CRD42022330706, PROSPERO [CRD42022330706].

## 1. Introduction

Severe acute respiratory syndrome coronavirus 2 (SARS-CoV-2), a novel coronavirus, is the causative agent of coronavirus disease 2019 (COVID-19). This disease was first reported at the end of 2019 in China (1) then rapidly spread across the world with multiple waves of different mutations of SARS-CoV-2 as variants of concern (VoC). As new, more transmissible variants emerge, vaccination rates increase and public health and social measures (PHSM) to mitigate transmission are eased over the course of the pandemic (e.g., density limits, lockdowns), it is important to understand if individual level risk factors for infection also change, so public health responses can prioritize those who are most at risk for infection and severe disease.

Social, environmental, and economic factors are known to influence SARS-CoV-2 infection risk in different populations (2–4). Longstanding structural drivers of health inequities have been highlighted, as there has been a disproportionate impact on underserved or disadvantaged community groups (3). Front-line essential workers were required to go on site throughout lockdowns. This created a greater risk of infection for those individuals compared with people who were able to work remotely from home (2). Individuals who are in precarious employment, reside in overcrowded or multi-generational housing, have lower education or income levels have generally been impacted more severely by the COVID-19 pandemic with higher risks for infection (5). People employed in roles which were unable to work from home or relied on cash in hand positions to support their families were more greatly impacted by PHSMs like lockdowns; these measures either resulted in loss of income or a disproportionate increase in risk of infection due to continued front line work (3, 5). The impact can be substantial, as many lower to medium income countries have high proportions of their population working in these types of employment; in parts of Africa, for example, more than 70% of the workforce is in informal employment (5, 6).

As vaccination rates increased, many countries eased PHSM. Easing of PHSM, along with immune evasion and therefore less vaccine effectiveness against infection for more recent variants, are likely to have changed who is most at risk of infection over time. Continuing to understand differences in risk factors between VoC as they emerge is important to inform public health responses and to strive for more equitable health outcomes, particularly early in a pandemic. Previous reviews have described specific disparities in incidence or severe outcomes from SARS-CoV-2 infection such as race/ethnicity, socioeconomic status, age, or sex (7–9). However, there have been no systematic literature reviews on risk for SARS-CoV-2 infection described by VoC. Understanding if risk factors change over time is valuable to add to the existing knowledge around the evolution of the COVID-19 pandemic. As we move into the phase of long-term public health management, compared with a short-term emergency response, findings from this review will be beneficial for informing future respiratory virus outbreak responses. As lockdowns were eased and restrictions lifted across many countries, we hypothesize that the risk factors for SARS-CoV-2 infection changed over time and as the Omicron variant was so highly transmissible, that most people were infected during this period. As there was increased community transmission, all people were at high risk of infection. The aim of this systematic literature review is to summarize the risk factors of SARS-CoV-2 infection in all age groups, by variant of concern, and to describe the effect PHSM may have had on the findings, where the data allow.

## 2. Methods

The protocol for this review was registered with PROSPERO (Prospero ID: CRD42022330706). This review was conducted following the guidelines from the Preferred Reporting Items for Systematic reviews and Meta-Analyses (PRISMA) statement (10).

### 2.1. Search strategy

A literature search was conducted for peer-reviewed published articles describing associations between sociodemographic, economic, or environmental risk factors and infection with SARS-CoV-2 in any human population. The search was restricted to articles in English from January 1, 2020, to May 5, 2022. Three electronic databases were searched: MEDLINE, PubMed and Embase. The search strategy outlined in Supplementary material 1 was utilized to extract references from these databases via the Ovid platform. Keywords included SARS-CoV-2, COVID-19, risk (s), infection, socioeconomic, sociodemographic, geographic, employment, social determinants of health and population density.

### 2.2. Selection criteria

This systematic review included studies which assessed risk factors associated with SARS-CoV-2 infection and reported these associations as odds ratios, hazard ratios, prevalence ratios or incidence risk ratios for any age in a human population. Each risk factor identified in these studies was required to have one of these corresponding measures and 95% confidence intervals for inclusion. Studies included were limited to cohort, cross-sectional and case-control studies. The following study designs were excluded: randomized controlled trials, case series and reports, ecological studies, qualitative studies, systematic reviews, and opinion-based articles. Studies that measured illness severity or mortality were excluded from the review unless they also included risk factors for infection independently, with extractable data for the relevant outcome. Any articles that only included animal studies were also excluded.

The outcome of interest for inclusion was SARS-CoV-2 infection, laboratory confirmed via reverse transcription polymerase chain reaction (RT-PCR) testing, rapid antigen self-testing (RAT), lateral flow tests or a neutralizing antibody test. Diagnosis through clinical presentation and meeting symptom criteria were excluded unless there was subsequent laboratory confirmation.

### 2.3. Article screening

Search results from the three databases were imported and stored in Covidence systematic review software (https://www.covidence.org). This software was also utilized for the removal of duplicate articles and coordination of the title and abstract and full text screenings. A two-stage review process was completed, with the first screening including a title and abstract screening conducted based on the outlined inclusion criteria. A full text review of papers eligible for inclusion was completed as the second stage process.

To limit the risk of bias in the study selection process, 10% of the total studies returned in the database searches were title and abstract screened by a second independent researcher (OB). All articles in the full text review were screened by both reviewers (MH and OB) for final inclusion in the review. Any disagreements during the selection process were resolved via discussion between the two reviewers. A third-party researcher was available to settle any unresolved points of contention if they presented through the study selection process.

### 2.4. Data extraction and synthesis

Data extracted from each included study were: geographic location of the study, study design, study population, sample size/case numbers, study period/timeframe, testing method for confirmation of SARS-CoV-2 infection, variant or variant subgroup if reported, risk factor or exposure, results (measure and 95% confidence interval) and the criteria for testing (e.g., recruited in community centers or tested upon symptomatic presentation to health centers). Only factors which were positively or negatively associated with SARS-CoV-2 infection were extracted. Where papers did not specify which VoC they were investigating, the variant for that study was determined using the study time frame and the dominant variant circulating in that location during the date range, as specified through the WHO online resources or other government websites as needed.

To determine study quality, a risk of bias assessment was conducted for each of the included studies using the Risk of Bias in Non-randomized Studies of Exposure (ROBINS-E) assessment tool. The data was synthesized using a narrative summary with respect to the quality of study and risk of bias in each study and any associations which can be made.

Due to the high level of heterogeneity in the study design, risk factors, study participant populations and the measures of associations, meta-analysis was not possible. The information gathered from each paper was instead synthesized in a tabular format and interpreted through narrative analysis.

## 3. Results

**Figure 1:**
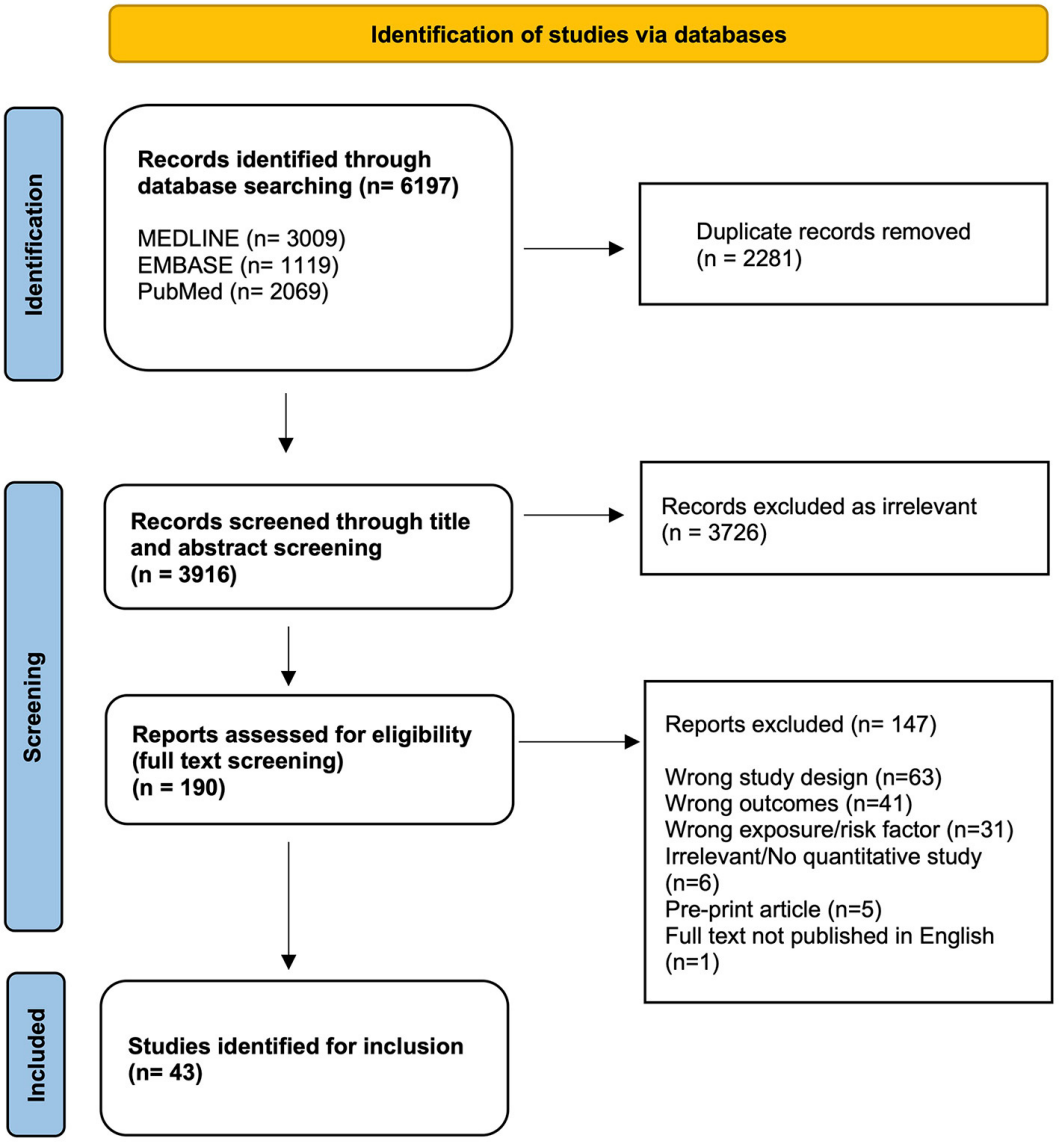
PRISMA flow diagram of study selection process and results.

Searches conducted in three online databases returned 6,197 results. During screening, 2,281 duplicate records were removed, and 3,916 results were title and abstract screened. The full text review resulted in 43 articles being eligible for inclusion (Figure 1).

The study characteristics and extracted data from included studies are shown in Supplementary Table 1. There were seven case control studies, 22 cohort studies and 14 cross-sectional studies. Studies were conducted in the United States of America (USA), United Kingdom, Spain, France, South Korea, Norway, Italy, Portugal, Angola, Indonesia, Colombia and varied between country-wide studies and smaller, regional populations. The total number of participants in the 43 studies was 34,818,271 but case numbers varied considerably between studies.

Several social, economic, and environmental risk factors for SARS-CoV-2 infection were identified (Table 1). The most frequently identified risk factors were ethnicity/race (23 studies), socioeconomic status/strata (SES) (19 studies), age (17 studies), increased household size (11 studies), comorbidities (11 studies), occupation nine studies), and sex/gender (nine studies). Several studies utilized retrospective data from centralized health surveillance systems or biobanks which contained demographic data of interest which was then linked to SARS-CoV-2 testing data. The remaining studies issued self-reporting questionnaires to the study population via email or phone interview or collected survey questions upon testing on location.

**Table 1:** Identified risk factors for SARS-CoV-2 infection and corresponding frequency in selected studies (n = 43).

**Risk factor categories**	**Number of studies reporting a positive association with infection**
Minority ethnicity/race/country of birth	23
Lower socioeconomic status	19
Older age	17
Increased household size/cohabitants and type of housing	11
Presence of comorbidities/health status	11
Male gender	9
Occupation	9
Lower income level/occupation status	8
Obesity	5
Lower education level	5
Residential factors—rural/urban, population size	4
Employment factors—inability to WFH, form of commute, in-person meetings	4
Presence of mental disorders/psychiatric diagnosis	2
Environmental—lack of access to safe drinking water, increased air pollution	2
Smoking status	2

The prominent VoC was recorded for each study and the number of studies conducted per VoC are listed in Table 2. There were 33 studies which were conducted during the predominance of the ancestral strain of SARS-CoV-2 (11–43). Through the period of 2020-early 2021, while the ancestral and Alpha strains were circulating, six studies collected data (44–49); two studies were conducted for an extended period which included ancestral, Alpha and Delta circulating (50, 51); one study collected data entirely during Alpha predominance (52); and one study while Delta was the prominent variant (53).

**Table 2:** Included studies by VoC.

**Year (s) of study**	**Dominant VoC circulating during study**	**Number of studies**
2020	Ancestral	33
2020–Early 2021	Ancestral and Alpha	6
2020–Mid 2021	Ancestral, Alpha, and Delta	2
Early 2021	Alpha	1
Mid 2021	Delta	1

### 3.1. Risk of bias assessment

The ROBINS-E tool was completed for each selected study. Of the 43 studies, 90.7% (39 studies) reported at least one minor concern of bias in the study design so were given an overall moderate risk of bias allocation. Three studies were rated as having low risk of bias overall (14, 18, 50) and one study was rated as high risk of bias, due to a lack of adjustment for confounding factors (34). Most studies found “some concern” in domain 3, relating to participant selection and risks of selection bias.

### 3.2. Risk factors for SARS-CoV-2 infection

Ethnicity or race or country of birth was positively associated with increased risk of SARS-CoV-2 infection in 23 studies and like most other risk factors, there was significant heterogeneity in these findings. The ethnic minority groups which were reviewed varied considerably between studies, due to the different regions each study was conducted in. Studies from the USA commonly explored Hawaiian/Pacific Island, Hispanic or Latinx communities (30, 31, 36, 37, 41, 43) where conversely, studies conducted through Europe, would investigate other European nations or African regions (21, 28, 46). One study also reviewed the use of non-English language in the family/home and found that the use of non-English language increased infection risk (41).

SES was listed as a risk factor for 19 studies and there was significant heterogeneity in the methods that studies took to measure SES. Several studies utilized measures of deprivation of geographical areas or communities to indicate an individual's SES; the Townsend deprivation score (26, 27, 51) and index of multiple deprivation (24, 48) were two commonly defined measures utilized. Other studies measured socioeconomic status using self-defined criteria (criteria included, but not limited to, income, education level or residential location) (17). Nevertheless, in all studies which investigated the association between SES and SARS-CoV-2 infection, there was shown to be a strong positive association with lower socioeconomic status or deprivation measure and an increased risk of infection.

Age was analyzed in 17 selected studies. There were some conflicting findings between studies, but for most, the findings indicated that older age had a positive association with higher risk of infection. There was one study where the 15–29-year-old age range showed an increased risk of infection when compared with the 50–59-year-old age bracket (21). Increase in age did not always have a linear increase in infection risk e.g., overall older age had higher infection risk but one study, conducted during ancestral strain predominance, showed 18–64- year-old and 75+ both had higher risk of infection than 65–75-year-old, when compared to a 0–17-year-old group (24).

There were several studies which investigated the link between household size and infection with SARS-CoV-2 and all concurred that as the household size increases, so does the risk for infection (12, 13, 19, 23, 50, 53). There was one study which investigated the type of housing and risk of infection and found that living in a shelter/social housing was associated with increased risk (53). Two studies explored the impact of living in an aged care facility on risk of infection and found an increased likelihood of infection (19, 21). One study conducted in France also found that the activities of the other individuals in the household, influenced another individuals risk of infection; specifically, living in a household with children who attended school or had a childminder (12).

Some studies explored associations with infection risk from specific jobs or employment sectors (like healthcare workers, aged care workers or shift work) and found some frontline, healthcare, and hospitality jobs to have increased risk of infection (15, 21, 44). Several studies also looked at the income level and the association with infection; income ranges varied throughout studies, but all showed associations between lower income and increased infection risk (17, 18, 20, 23, 32, 40, 50). These studies often also compared this with unemployment and found that retirement or unemployment increased the risk of infection. One study investigated an individual's employment status but also the employment status of their partner, and found that the unemployed status of their partner was associated with an increase in risk of infection (14). One study investigated the different modes of working (remote, on site, hybrid etc.) to determine the risk for each form of work (53). This found that when compared to working full time at the office on site, a hybrid option served as a protective factor and reduced risk of infection. All other forms of work had no association to infection. Unemployment/not working at all did however have an increased risk of infection compared to full time office work (53). Lower levels of education was also identified as a risk factor in multiple studies (19, 23, 28, 51), finding that risk of infection decreased the higher the level of education attained.

There were several other individual risk factors which broadly fell into environmental factors. One study found that not having access to safe drinking water increased the risk of infection (22). Similarly, one study also found that being a current or previous smoker also had an increased infection risk (29). There was one study which found that increased air pollution levels was a risk factor for infection (48).

The comorbidities investigated in these studies included diabetes (17, 41, 51), major depressive disorder (52), moderate to severe disability (18), respiratory disease, cerebrovascular disease, and hypertension (51). Diabetes was the most common comorbidity to show a positive association to infection across multiple studies. Diagnosed mental health and psychiatric disorders were also explored and showed that an individual with a mental disorder is at a greater risk of infection (51). The study exploring psychiatric disorders however, only showed a positive association with major depressive disorder whereas all other disorders explored (e.g., schizophrenia) showed no association (52).

## 4. Discussion

This systematic literature review identified several risk factors associated with having SARS-CoV-2 infection across multiple studies, including minority ethnicity, older age, male gender, lower SES, increased household size, occupation, presence of comorbidities and lower income level. Most included studies were conducted early in the pandemic so changes to risk factors that may have occurred with the changes in VoC over time could not be determined. There are two studies which were conducted during the Alpha (52) and Delta (53) periods of circulation but both of these studies reported on unique risk factors which were not included in other studies. Notably, the study by Grant et al. (53) in France, performed an analysis comparing the risk factors between Delta and Ancestral strain SARS-CoV-2, using genomic testing and found no difference in the risk factors between Delta or Ancestral strain variants. This was the only study directly comparing risk factors by VoC. Further studies assessing the risk factors during Delta, Omicron or any future variants may be beneficial, although during the period of Omicron predominance, most people were at risk of infection due to the high transmissibility of this VoC. While this review did not identify any studies of more recent VoC to determine any change to risk factors over time, it does provide further evidence of clear risk factors for ancestral, Alpha and Delta VoC. This will add to the substantial knowledge base of risk factors for viral infections in the early stages of a pandemic or outbreak.

The evolution of the pandemic and the changes to PHSM as governments eased restrictions also has an impact on risk of infection. Some studies did refer to the impact of government restriction changes and testing capacity improvements on their results (15, 20, 53). The study by Magnusson in Norway (15), highlighted the shift in risk of infection by occupation, as the stages of PHSM changed. The beginning of the first wave saw police officers, firefighters, healthcare workers and taxi/bus drivers with the greatest risk but as the second wave was recorded, the highest risk of infection had moved to frontline and entertainment occupations like bar tenders and retail workers. This highlights the evolution of risk as public health interventions change over time and the risk shifts to different parts of the community. Studies such as these should be utilized to inform future public health strategies, in particular vaccination scheduling in order of greatest infection risk and in anticipation of the changes in PHSM and risk.

A similar study performed in Spain, reviewed changes in risk of infection by age and socioeconomic status (20). The three waves noted in this study identified older individuals in aged care facilities being the most impacted in the first wave, with highest risk. The second wave, also correlated with wider testing capabilities, and showed a significant change where low SES individuals were at higher risk. The return of the seasonal fruit pickers also aligned with that second wave in Spain; this cohort was identified as particularly vulnerable as they are often migrants from other low- and middle-income countries returning to Spain for work. As PHSMs were altered, subsequent waves occurred in different naïve populations of the community.

Due to the heterogeneity between specific risk factor definitions in each individual study, some risk factors, such as SES, could only be broadly summarized. Some studies measured using a series of indicators (like education level and income) whereas other studies chose to use measures of deprivation as an indicator of socioeconomic strata. The index of multiple deprivation was one such measure which combines seven different domains from the indices of deprivation as created by the UK government to reflect areas of the nation and their cumulative level of deprivation (54). This measure was utilized by three studies completed in the United Kingdom (24, 47, 48). Another similar measure, the Townsend deprivation score, was also used by multiple studies to measure deprivation and SES (25–27, 51). While these measures may not be directly comparable across studies, their overall findings present concordant results. All studies which measured SES reported conclusively that the more significant the deprivation or lower SES bracket, the greater risk of infection.

Several individual risk factors can also be related with other identified risk factors; for example, income level and employment status, both of which were individually identified as risk factors, also form part of defining an individual's SES grouping. It is important to better understand the links between different risk factors and the behaviors that drive them so targeted responses and interventions can be designed. This relationship between risk factors was also observed in the most commonly reported risk factors, minority ethnicity or race and country of birth (18, 28, 30, 31, 36, 37, 41, 43, 46). As previously highlighted, one study found that speaking a language other than English in the household, was an indicator of increased risk of infection (41). While this risk factor may have only been identified in a single study, it likely reflects the increased risk of infection identified in ethnic minority and lower socio-economic groups and those that could not work from home. Retirement or unemployment status was also found to be a risk factor for infection in several studies. Some studies have shown that transmission in households and other residential settings (like aged care facilities) are major drivers of SARS-CoV-2 infection (55, 56). As retirement and unemployment may also be associated with living in settings like aged care facilities or larger households, these may be the factors that put these groups of individuals at higher risk of infection. This also supports the common finding of increased household size being a risk factor for infection. From these studies we can clearly identify that there is a disproportionate burden of risk for these groups, therefore it is important as PHSMs are rolled back and new variants emerge, ongoing research is conducted to monitor any changes in risk. This will assist with informing future public health responses to COVID-19 or another pandemic, and long-term policy to support equitable health outcomes.

Our review has a number of limitations. Firstly, the risk of bias assessment only identified one study which had high risk of bias. However, as identified through the risk of bias assessment, there may be potential biases introduced in the selection of participants in multiple studies. Observational studies investigating risk factors for SARS-CoV-2 infection can often be subject to selection bias, in both directions (57). Due to government and public health responses, testing was limited and highly specified during most of 2020. As most of the included studies were conducted in 2020, the testing criteria were often narrow and commonly restricted to healthcare workers or symptomatic hospital presentations. This limited testing may introduce bias as asymptomatic presentations or individuals who were not able to present to a hospital for care may not have been recorded, leading to associations less representative of the wider population. Studies which are conducted when testing criteria were broadened, or if there was more comprehensive surveillance testing to capture asymptomatic infections, may have reduced impact of this bias. Secondly, another consideration is the exclusion of gray literature and pre-print material, and this may have biased the studies to the earlier VoC. Thirdly, the review mostly consists of studies from high income countries, with only three studies from low- and middle-income countries. Health inequity is much greater in low- and middle-income countries and understanding the changes to risk in these settings may be particularly beneficial to guide their public health responses to communicable diseases. This warrants further investigation to identify whether risk factors change or are disproportionately more severe in LMIC when compared to higher income countries. It should also be noted that different COVID-19 vaccine formulations have different effects on infection rates. Vaccine effectiveness also differs by variant and by immunity derived from prior infection, although studies included in this review did not describe how vaccines modified the risk of infection. The majority of included studies were conducted prior to the introduction of COVID-19 vaccines, so the potential effects of this would only impact few studies, nevertheless, this is a limitation of our findings.

With the recent decision by the WHO that the COVID-19 pandemic no longer constitutes a Public Health Emergency of International Concern (PHEIC) and better reflects a position of long-term public health management; (58) the findings of this review contribute to the collective knowledge of risk factors for infection with respiratory viruses in the early stages of a pandemic. The information presented here should be useful for PHSM and policy decision making in future outbreaks of novel respiratory viruses, helping ensure that the groups who are at greatest risk are appropriately protected, where possible, and prioritized in vaccine rollouts.

In conclusion, this systematic review found clear risk factors for SARS-CoV-2 infection during transmission of the ancestral strain. Further studies including risk factors during predominance of Delta, Omicron and future variants would be important to inform current and future long-term public health responses. Identifying how risk changes as new VoC emerge, the impact of VoC on immune evasion and how risk may change as PHSM are adjusted, allows for a better understanding of who is at greater risk. This may support development of strategies that target priority groups at different stages of a pandemic and, informing vaccination strategies and other interventions that may reduce transmission. Understanding this change in risk over time may also be beneficial to inform public health responses for future outbreaks of other novel infectious diseases as they evolve during a pandemic.

## Data availability statement

The original contributions presented in the study are included in the article/Supplementary material, further inquiries can be directed to the corresponding author.

## Author contributions

MH, JH, and FR contributed to the conception and design of the study. MH drafted the review protocol, executed the search strategy, and wrote the first draft of the manuscript. JH and FR reviewed and contributed to the protocol. MH and OB screened and reviewed all titles, abstracts, and included studies. JH, OB, and FR critically reviewed and wrote sections of the manuscript multiple times. All authors contributed to manuscript revision, read, and approved the submitted version.
