# Supplementary material for: Risk factors for SARS-CoV-2 infection during the early stages of the COVID-19 pandemic: a systematic literature review
Source: Front Public Health. 2023 Jul 31;11:1178167. doi: 10.3389/fpubh.2023.1178167 (PMC10424847; doi:10.3389/fpubh.2023.1178167)
Supplement: Supplementary file 1 [file Data_Sheet_1.PDF]

Supplementary Material S1

Database search strategies – MEDLINE, PubMed and Embase.
Search completed on 5 May 2022

MEDLINE – 3009 results	
**N**	**Search Queries**
1	COVID-19/ or SARS-CoV-2/
2	covid-19 testing/ or covid-19 serological testing/ or covid-19 nucleic acid testing/
3	(2019-novel or 2019nCoV or 2019-nCoV or COVID-19 or COVID19 or COVID-2019 or COVID2019 or CONVID-19 or CONVID19 or CORVID-19 or CORVID19 or CoV2 or CoV-2 or HCoV* or Ncov* or Ncorona* or Ncorono* or NcovChina* or NcovChinese* or NcovHubei* or NcovWuhan* or SARS2 or SARS-2 or SARScoronavirus2 or SARScoronavirus-2 or SARScoronovirus2 or SARScoronovirus-2 or SARSCov19 or SARSCov-19 or SARS-CoV-2 or SARSCoV-2 or SARSCoV2 or WN-CoV or WNCoV or wuhan-virus).tw,kf,ot.
4	((pneumonia* or outbreak* or respiratory-illness* or respiratory-disease* or respiratory-symptom* or seafood-market* or food-market* or wildlife) and (Wuhan or China or Chinese or Hubei or Huanan)).tw,kf,ot.
5	((new or novel or nouveau or "19" or "2019" or Wuhan or Hubei or Huanan or China or Chinese) adj3 (coronavirus* or corona virus* or betacoronavirus* or CoV or HCoV)).tw,kf,ot.
6	(coronavirus/ or betacoronavirus/ or coronavirus infections/) and (disease outbreaks/ or epidemics/ or pandemics/)
7	((coronavirus* or corona-virus* or betacoronavirus*) adj3 (pandemic* or epidemic* or outbreak* or crisis)).tw,kf,ot.
8	1 or 2 or 3 or 4 or 5 or 6 or 7
9	((risk or risks) and infection*).tw,kf,hw.
10	sociodemographic factors/ or exp socioeconomic factors/
11	(ses or socioeconomic* or economic* or employ* or unemploy* or sociodemographic* or socio-demographic* or geographic* or postcode* or zipcode* or zip-code*).tw,kf.
12	"social determinants of health"/ or population density/
13	10 or 11 or 12
14	8 and 9 and 13
15	limit 14 to (english language and yr="2020 -Current")
16	limit 15 to (case reports or comment or editorial or guideline or letter or practice guideline or preprint)
17	15 not 16

Embase – 1119 results	
**N**	**Search Queries**
1	coronavirus disease 2019/ or SARS coronavirus/ or experimental coronavirus disease 2019/ or exp severe acute respiratory syndrome coronavirus 2/
2	covid-19 testing/ or covid-19 nucleic acid testing/ or covid-19 serological testing/
3	(2019-novel or 2019nCoV or 2019-nCoV or COVID-19 or COVID19 or COVID-2019 or COVID2019 or CONVID-19 or CONVID19 or CORVID-19 or CORVID19 or CoV2 or CoV-2 or HCoV* or Ncov* or Ncorona* or Ncorono* or NcovChina* or NcovChinese* or NcovHubei* or NcovWuhan* or SARS2 or SARS-2 or SARScoronavirus2 or SARScoronavirus-2 or SARScoronovirus2 or SARScoronovirus-2 or SARSCov19 or SARSCov-19 or SARS-CoV-2 or SARSCoV-2 or SARSCoV2 or WN-CoV or WNCoV or wuhan-virus).tw,kf,dq,ot.
4	((pneumonia* or outbreak* or respiratory-illness* or respiratory-disease* or respiratory- symptom* or seafood-market* or food-market* or wildlife) and (Wuhan or China or Chinese or Hubei or Huanan)).tw,kf,dq,ot.
5	((new or novel or nouveau or "19" or "2019" or Wuhan or Hubei or Huanan or China or Chinese) adj3 (coronavirus* or corona virus* or betacoronavirus* or CoV or HCoV)).tw,kf,dq,ot.
6	(coronavirinae/ or betacoronavirus/ or coronavirus infection/) and (epidemic/ or pandemic/)
7	((coronavirus* or corona-virus* or betacoronavirus*) adj3 (pandemic* or epidemic* or outbreak* or crisis)).tw,kf,dq,ot.
8	severe-acute-respiratory-syndrome-coronavirus-2.hw.
9	coronavirus-disease-2019.hw.
10	1 or 2 or 3 or 4 or 5 or 6 or 7 or 8 or 9
11	((risk or risks) and infection*).tw,kf,hw,dq.
12	socioeconomics/ or exp economic status/ or exp income group/ or exp poverty/ or exp socioeconomic distribution/
13	"social determinants of health"/
14	population density/
15	12 or 13 or 14
16	10 and 11 and 15
17	limit 16 to (english language and yr="2020 -Current")
18	case report/
19	limit 17 to (conference abstract or conference paper or "conference review" or editorial or letter or "preprint (unpublished, non-peer reviewed)")
20	17 not (18 or 19)

PubMed – 2069 results	
**N**	**Search Queries**
#1	Title/Abstract<br>“2019-novel” OR “2019nCoV” OR “2019-nCoV” OR “COVID-19” OR “COVID19” OR “COVID-2019” OR “COVID2019” OR “CONVID-19” OR “CONVID19” OR “CORVID-19” OR “CORVID19” OR “CoV2” OR “CoV-2” OR “HCoV*” OR “Ncov*” OR “Ncorona*” OR “Ncorono*” OR “NcovChina*” OR “NcovChinese*” OR “NcovHubei*” OR “NcovWuhan*” OR “SARS2” OR “SARS-2” OR “SARScoronavirus2” OR “SARScoronavirus-2” OR “SARScoronovirus2” OR “SARScoronovirus-2” OR “SARSCov19” OR “SARSCov-19” OR “SARS-CoV-2” OR “SARSCoV-2” OR “SARSCoV2” OR “WN-CoV” OR “WNCoV” OR “wuhan-virus”
#2	Title/Abstract<br>(“pneumonia*” OR “outbreak*” OR “respiratory-illness*” OR “respiratory-disease*” OR “respiratory-symptom*” OR “seafood-market*” OR “food-market*” OR “wildlife”) AND (“Wuhan” OR “China” OR “Chinese” OR “Hubei” OR “Huanan”)
#3	Title/Abstract<br>(“new” OR “novel” OR “nouveau” OR “19” OR “2019” OR “Wuhan” OR “Hubei” OR “Huanan” OR “China” OR “Chinese”) AND (“coronavirus*” OR “corona virus*” OR “betacoronavirus*” OR “CoV” OR “HCoV”)
#4	Title/Abstract<br>(“coronavirus*” OR “corona-virus*” OR “betacoronavirus*”) AND (“pandemic*” OR “epidemic*” OR “outbreak*” OR “crisis”)
#5	#1 OR #2 OR #3 OR #4
#6	title/abstract<br>(“risk” OR “risks”) AND “infection*”
#7	Title/Abstract<br>“ses” OR “socioeconomic*” OR “economic*” OR “employ*” OR “unemploy*” OR “sociodemographic*” OR “socio-demographic*” OR “geographic*” OR “postcode*” OR “zipcode*” OR “zip-code*” OR "economic-status” OR "poverty” OR "social-determinant*" OR "population-density"
#8	All fields<br>*NOTNLM OR publisher[sb] OR inprocess[sb] OR pubmednotmedline[sb] OR indatareview[sb] OR pubstatusaheadofprint*
#9	#5 AND #6 AND #7 AND #8
Years limited to 2020 onward and limited to English	
