# Supplementary material for: Risk factors for SARS-CoV-2 infection during the early stages of the COVID-19 pandemic: a systematic literature review
Source: Front Public Health. 2023 Jul 31;11:1178167. doi: 10.3389/fpubh.2023.1178167 (PMC10424847; doi:10.3389/fpubh.2023.1178167)
Supplement: Supplementary file 2 [file Table_1.pdf]

Supplementary Table 1. Summary of extracted data of included studies (n=43)

Study ref	Country, nation/ city	Study design	Study population	Number of participants	Study time frame	Lab method for SARS-CoV-2 confirmation	Testing criteria	Variant(s)/ variant subgroup(s)	Measured risk factor(s)/ exposure(s)	Measure of association	Factors positively associated with SARS-CoV-2 infection	Factors negatively associated with SARS-CoV-2 infection
**(1)**	Angola, Luanda	Cross-sectional study	Individuals tested upon presentation to hospital	622	Jan 2020 - Sep 2020	RT-PCR	Symptomatic, close contact or returned traveller	Ancestral	Age, resident of Luanda province	AOR	<10 years: aOR 1 (ref)<br>20-29 years: aOR 9.78 (95% CI, 2.13-44.9)<br>30-39 years: aOR 11.9 (95% CI, 2.30-52.2)<br>40-49 years: aOR 9.23 (95% CI, 2.01-42.3)<br>50-59 years: aOR 14.7 (95% CI, 3.20-67.2)<br>>60 years: aOR 23.3 (95% CI, 4.83-112)<br><br>Luanda province: aOR 7.40 (95% CI, 1.64-33.4)	
**(2)**	Colombia, Bogota	Cross-sectional study	Healthcare workers, frontline workers and Individuals recruited through the CoVIDA contact tracing strategy	58,638	Mar 2020 – Apr 2020 and Sep 2020 – Dec 2020	RT-PCR	Symptomatic or mildly symptomatic individuals with no history of a positive test recruited into the study matching the target study population	Ancestral/Alpha	Age, cohabitants, occupation, socioeconomic strata	OR	>60 years old: 1 (Ref)<br>18-29 years old: aOR 1.22 (95% CI, 1.05-1.41)<br><br>>3 habitants in a house: aOR 1.27 (95% CI, 1.17-1.36)<br><br>Low-low SES: aOR 3.91 (95% CI, 2.86-5.35)<br>Low SES: aOR 2.55 (95% CI, 1.91-3.40)<br>Middle-low SES: aOR 2.33 (95% CI, 1.75-3.09)<br>Middle SES: aOR 1.37 (95% CI, 1.02-1.85)<br><br>Front-line occupation (police, firefighter): aOR 2.27 (95% CI, 1.80-2.86)<br>Construction workers: aOR 1.90 (95% CI, 1.35-2.68)<br>Essential office work: aOR 1.37 (95% CI, 1.17-1.61)<br>Public/private driver: aOR 1.43 (95% CI, 1.17-1.74)<br>Other occupations (e.g., actor, musicians): aOR 1.28 (95% CI, 1.07-1.52)<br>Informal employment: aOR 1.61 (95% CI, 1.34-1.93)	

Study ref	Country, nation/ city	Study design	Study population	Number of participants	Study time frame	Lab method for SARS-CoV-2 confirmation	Testing criteria	Variant(s)/ variant subgroup(s)	Measured risk factor(s)/ exposure(s)	Measure of association	Factors positively associated with SARS-CoV-2 infection	Factors negatively associated with SARS-CoV-2 infection
**(3)**	France	Case control	Cases who tested positive anywhere within France and controls who were matched by demographic to cases from a market research company	5,139	Oct 2020 - Nov 2020	RT-PCR	Not specified	Ancestral	Children in household and their current education level, household size, employment category, in-person work meeting, carpooling	aOR	Number of persons in the household:<br>2: OR 1.17 (95% CI, 1.04-1.31)<br>3: OR 1.38 (95% CI, 1.21-1.58)<br>4: OR 1.60 (95% CI, 1.35-1.87)<br>5: OR 1.81 (95% CI, 1.44-2.25)<br>6+: OR 2.33 (95% CI, 1.70-3.13)<br><br>Children in the house who attended:<br>- Day-care: aOR 1.31 (95% CI, 1.02-1.62)<br>- Kindergarten: aOR 1.27 (95% CI, 1.09-1.45)<br>- Middle school: aOR 1.30 (95% CI, 1.15-1.47)<br>- High school: aOR 1.18 (95% CI, 1.05-1.34)<br><br>Profession type:<br>Independent profession: OR 1.75 (95% CI, 1.46-2.08)<br>Senior Executive: OR 1.27 (95% CI, 1.15-1.42)<br>Worker: OR 1.83 (95% CI, 1.57-2.13)<br><br>In-person professional meeting: OR 1.15 (95% CI,1.04-1.26)<br>Carpooling: OR 1.47 (95% CI, 1.28-1.69)	Children in the house who attended:<br>- Primary school: aOR 0.87 (95% CI, 0.77-0.99)<br><br>Professional activity:<br>Office work without teleworking: aOR 1 (ref)<br>Not working: aOR 0.56 (95% CI, 0.48-0.65)<br>Working but no office work: aOR 0.86 (95% CI, 0.76-0.96)<br>Office work with partial teleworking: aOR 0.76 (95% CI, 0.66-0.87)<br>Office work with complete teleworking: aOR 0.65 (95% CI, 0.56-0.75)

Study ref	Country, nation/ city	Study design	Study population	Number of participants	Study time frame	Lab method for SARS-CoV-2 confirmation	Testing criteria	Variant(s)/ variant subgroup(s)	Measured risk factor(s)/ exposure(s)	Measure of association	Factors positively associated with SARS-CoV-2 infection	Factors negatively associated with SARS-CoV-2 infection
**(4)**	France	Case control	Cases who tested positive anywhere within France and controls who were matched by demographic to cases from a market research company.	18,194	May 2021 - Aug 2021	RT-PCR	Not specified	Delta	Type of public transport, housing type, cohabitants, employment/profession type	OR	Professional category:<br>Senior executive: aOR 1.9 (95% CI, 1.7-2.2)<br>Intermediate profession: aOR 1.3 (95% CI, 1.2-1.5)<br>Worker, farmer: aOR 1.2 (95% CI, 1.1-1.4)<br>Student: aOR 1.6 (1.3-1.9)<br><br>Carpooling: aOR 1.3 (95% CI, 1.2-1.4)<br>Taxi: aOR 1.5 (95% CI, 1.2-1.8)<br>Subway: aOR 1.2 (95% CI, 1.0-1.4)<br>National train: aOR 1.3 (95% CI, 1.1-1.6)<br>Aeroplane: aOR 1.7 (95% CI, 1.3-2.2)<br><br>Shelter or social housing: aOR 2.3 (95% CI, 1.2-4.3)<br><br>Having a child who is looked after by a childminder: aOR 1.6 (95% CI, 1.3-2.0)	Professional category:<br>Retired: aOR 0.5 (95% CI, 0.4-0.7)<br>Unemployed: aOR 0.5 (95% CI, 0.4-0.6)
**(5)**	Indonesia, Jakarta	Cross-sectional study	Health and non-healthcare workers in hospitals	1,397	Apr 2020 - Jul 2020	RT-PCR	Healthcare staff with symptoms or close contact	Ancestral	Occupation, household size	OR	Healthcare worker: aOR 8.31 (95% CI, 1.27-54.54)<br><br>Household size >5: aOR 4.09 (95% CI, 1.02-16.43)	Middle- and upper-class hospital workers: aOR 0.06 (95% CI, 0.01-0.66)
**(6)**	Italy	Cross-sectional study	Pregnant women admitted for pregnancy-related health care	896	Mar 2020 - Apr 2020	RT-PCR	Women admitted for pregnancy related care, asymptomatic testing	Ancestral	Ethnicity, age	OR	Ethnicity (non-Italian): aOR 2.15 (95% CI, 1.12-4.11)	Age, >35 years old: aOR 0.40 (95% CI, 0.21-0.78)

Study ref	Country, nation/ city	Study design	Study population	Number of participants	Study time frame	Lab method for SARS-CoV-2 confirmation	Testing criteria	Variant(s)/ variant subgroup(s)	Measured risk factor(s)/ exposure(s)	Measure of association	Factors positively associated with SARS-CoV-2 infection	Factors negatively associated with SARS-CoV-2 infection
**(7)**	Norway	Prospective cohort study	Entire population of Norway	3,579,608	Feb 2020 – Jul 2020 and Aug 2020 – Dec 2020	RT-PCR	Initially included having severe disease, being in an at-risk group or health personnel; this later changed to include everyone with symptoms or having been a close contact of a confirmed case	Ancestral	Occupation	OR	First wave:<br>Nurses, physicians, dentists, physiotherapists, bus, tram, or taxi drivers:<br>1.5-3 times the odds of infection.<br><br>Second wave:<br>Bartenders, transport conductors, travel stewards, waiters, and food service attendants:<br>1.5-2 times odds of infection.	
**(8)**	Norway	Cohort study	<18-year-olds	1,219,184	Mar 2020 - Nov 2021	RT-PCR and RAT	Symptomatic presentation to hospital	Ancestral/Alpha/D elta	Age, sex, household size, low family income, overcrowded housing, residential location, country of origin/birth	HR	Living in municipalities > 50,000 residents: aHR 1.61 (95% CI, 1.58 to 1.64)<br><br>Household size:<br><2: aHR 1 (ref)<br>4 people: aHR 1.14 (95% CI, 1.10-1.18)<br>5 people: aHR 1.26 (95% CI, 1.21-1.31)<br>>6 people:<br>aHR 1.53 (95% CI, 1.47-1.60).<br><br>Overcrowded living conditions:<br>aHR 1.27 (95% CI, 1.24-1.30)<br><br>Low family income: aHR 0.26 (95% CI, 1.23-1.30)<br><br>Female:<br>aHR, 1.01 (95% CI, 1.00-1.02)<br><br><6 years old: 1 (ref)<br>6 - 11 years old:<br>aHR 1.95 (95% CI, 1.90-1.99)<br>12-17 years old:<br>aHR 2.59 (95% CI, 2.52-2.65)<br><br>Country of origin:	

Study ref	Country, nation/ city	Study design	Study population	Number of participants	Study time frame	Lab method for SARS-CoV-2 confirmation	Testing criteria	Variant(s)/ variant subgroup(s)	Measured risk factor(s)/ exposure(s)	Measure of association	Factors positively associated with SARS-CoV-2 infection	Factors negatively associated with SARS-CoV-2 infection
											Nordic countries: aHR 1 (ref)<br>Europe: aHR 1.54 (95% CI, 1.51-1.58)<br>Latin America: aHR 1.21 (95% CI, 1.14-1.29)<br>Middle East and North Africa: aHR 2.07 (95% CI, 1.99-2.16)<br>Africa: aHR 2.22 (95% CI, 2.14-2.31)<br>Asia: aHR 1.73 (95% CI, 1.68-1.78)	
**(9)**	Portugal	Cross-sectional study	Individuals notified as suspected cases of SARS-CoV-2 infection	223,333	Feb 2020 - Jun 2020	RT-PCR	Symptomatic testing at health care service	Ancestral	Socioeconomic deprivation (European deprivation index)	PR	European deprivation index quartiles:<br>Q1 (least deprived): 1(ref)<br>Q2: PR 1.37 (95% CI, 1.19-1.58)<br>Q3: PR 1.48 (95% CI, 1.26-1.73)<br>Q4: PR 1.73 (95% CI, 1.47-2.04)<br>Q5 (most deprived): PR 2.24 (95% CI, 1.83-2.75)	
**(10)**	Portugal, Lisbon, and Targus Valley areas	Case control	Individuals who tested positive in the surveillance system and community controls recruited from a random dialling service.	1,875	Sep 2020 - Oct 2020	RT-PCR	Not specified	Ancestral	Education level, occupation, cohabitants in the same room	OR	Tertiary education: OR 1 (ref)<br><4 years education: 1.79 (95% CI, 1.33-2.42)<br>6 years: OR 3.85 (95% CI, 2.50-6.08)<br>9 years: OR 2.16 (95% CI, 1.61-2.90)<br>12 years: OR 2.23 (95% CI,1.78-2.81)<br><br>Living with one additional person:<br>aOR 1.47 (95% CI, 1.14-1.91)<br><br>Aged care employee: aOR 4.99 (95% CI, 1.30-33.08)	Remote working:<br>No (office working): aOR 1 (ref)<br>Yes: aOR 0.30 (95% CI, 0.22-0.42)
**(11)**	South Korea	Case control	Individuals with and without diabetes residing in South Korea	60,656	Jan 2020 - Apr 2020	RT-PCR	No testing criteria listed	Ancestral	Income levels, diabetes with insulin usage, age	OR	Diabetic with insulin use:<br>aOR 1.25 (95% CI, 1.03-1.53)<br><br>Low income (employee): OR 1.09 (95% CI, 1.03-1.17)	

Study ref	Country, nation/ city	Study design	Study population	Number of participants	Study time frame	Lab method for SARS-CoV-2 confirmation	Testing criteria	Variant(s)/ variant subgroup(s)	Measured risk factor(s)/ exposure(s)	Measure of association	Factors positively associated with SARS-CoV-2 infection	Factors negatively associated with SARS-CoV-2 infection
			and tested for COVID-19								Low income (self-employed): aOR 1.180 (95% CI, 1.081-1.288)<br><br>Age:<br>>80 years old: aOR 1 (ref)<br><20: aOR 1.469 (95% CI, 1.257-1.718)<br>20-29: aOR 1.553 (95% CI, 1.351-1.785)<br>30-39: aOR 1.578 (95% CI,1.361-1.831)<br>40-49: aOR 1.514 (95% CI, 1.313-1.747)<br>50-59: aOR 1.504 (95% CI, 1.312-1.724)<br>60-69: aOR 1.498 (95% CI, 1.307-1.717)<br>70-79: aOR 1.329 (95% CI, 1.153-1.531)	
**(12)**	South Korea	Cohort study	Individuals residing in South Korea and tested for COVID-19	122,040	Jan 2020 - Jun 2020	RT-PCR	Not specified	Ancestral	Income level, Charlson comorbidity index/level of disability, comorbidities	OR	Lowest income group: OR 1.19 (95% CI, 1.12-1.27)<br><br>Average income level (Q1: Lowest): aOR 1.10 (95% CI, 1.01-1.19)<br><br>Income decreases of 5% was linked to a 1% increase in risk.<br><br>Severe disabilities: OR 1.45 (95% CI, 1.26-1.67)<br><br>Charlson comorbidity index 2020, 1 point: aOR 1.19 (1.18-1.20)<br><br>Dementia: aOR 1.97 (95% CI, 1.72-2.25)<br>Peptic ulcer disease: aOR 1.27 (95% CI, 1.17-1.36)<br>Hemiplegia: aOR 2.36 (95% CI, 1.83-3.05)<br>Mild liver disease: aOR 1.90 (95% CI,1.77-2.03)	Age:<br>20-29: aOR 1 (ref)<br>40-49: aOR 0.81 (95% CI, 0.75-0.88)<br>50-59: aOR 0.69 (95% CI, 0.64-.74)<br>60-69: aOR 0.51 (95% CI,0.47-0.55)<br>70-79: aOR 0.36 (95% CI, 0.32-0.40)<br>80+: aOR 0.28 (95% CI, 0.24-0.32)

Study ref	Country, nation/ city	Study design	Study population	Number of participants	Study time frame	Lab method for SARS-CoV-2 confirmation	Testing criteria	Variant(s)/ variant subgroup(s)	Measured risk factor(s)/ exposure(s)	Measure of association	Factors positively associated with SARS-CoV-2 infection	Factors negatively associated with SARS-CoV-2 infection
											Chronic pulmonary disease: aOR 3.62 (95% CI, 3.42-3.83)<br>Congestive heart failure: aOR 2.40 (95% CI, 2.16-2.67)<br>Myocardial infarction: aOR 4.64 (95% CI, 4.00-5.38)<br>AIDS/HIV: aOR 3.39 (95% CI, 1.46-7.87)	
**(13)**	Spain, Aragon	Retrospective cohort study	Individuals who underwent testing in the Aragon region	357,989	Mar 2020 - Dec 2020	RT-PCR	Symptomatic or close contact testing	Ancestral	Age and income levels/employment	OR	*Women:*<br>Age:<br>15-44: aOR 1.51 (95% CI, 1.45-1.58)<br>45-64: aOR 1.61 (95% CI, 1.54-1.68)<br>65-79: aOR 1.57 (95% CI, 1.47-1.69)<br>> 80 years or older:<br>OR 2.50 (95% CI, 2.33-2.68)<br><br>Employment:<br>Employee, earning >$18,000: aOR 1 (ref)<br>Unemployed: OR 1.23 (95% CI, 1.15-1.32)<br>Employed, Earning <$18,000: OR 1.25 (95% CI, 1.21-1.30)<br><br>*Men:*<br>Age:<br>15-44: aOR 1.73 (95% CI, 1.66-1.81)<br>45-64: aOR 2.05 (95% CI, 1.96-2.14)<br>65-79: 1.92 (95% CI, 1.79-2.06)<br>80+: aOR 2.52 (95% CI, 2.34-2.72)<br><br>Employment:<br>Employee, earning >$18,000: aOR 1 (ref)<br>Employee, earning <$18,000: aOR 1.12 (95% CI, 1.08-1.16)	

Study ref	Country, nation/ city	Study design	Study population	Number of participants	Study time frame	Lab method for SARS-CoV-2 confirmation	Testing criteria	Variant(s)/ variant subgroup(s)	Measured risk factor(s)/ exposure(s)	Measure of association	Factors positively associated with SARS-CoV-2 infection	Factors negatively associated with SARS-CoV-2 infection
**(14)**	Spain	Prospective cohort	People covered by Navarre Health Service	643,757	Jul 2020 - Dec 2020	RT-PCR or RAT	Screening all symptomatic and asymptomatic patients	Ancestral	Age, country of birth, employment sector	RR	Age<br>50-59: 1 (ref)<br>15-29: aRR 1.28 (95% CI, 1.24-1.33)<br>30-49: aRR 0.96 (95% CI, 0.93-0.99)<br>60-69: aRR 0.75 (95% CI, 0.72-0.79)<br>70-79: aRR 0.64 (95% CI, 0.59-0.68)<br>80+: aRR 1.23 (95% CI, 1.14-1.33)<br><br>Nursing home residents: aRR 3.24 (95% CI, 2.98-3.53)<br><br>Healthcare workers: aRR 1.23 (95% CI, 1.14-1.33)<br><br>Born outside of Spain:<br>Latin America: aRR 2.08 (95% CI, 2.01-2.14)<br>Sub-Saharan Africa aRR 1.21 (95% CI, 1.10-1.32)<br>North Africa aRR 1.44 (95% CI, 1.36-1.53)	
**(15)**	Spain, Almeria	Cross-sectional study	Individuals from vulnerable groups living in three unfavourable areas in the province	746	Jun 2020 - Dec 2020	Rapid Neutralising Antibody test & RT-PCR	Recruitment into the study using selection criteria	Ancestral	No economic income, no access to safe drinking water	OR	No economic income: OR 2.26 (95% CI, 1.41-3.62)<br><br>No access to safe drinking water:<br>OR 9.23 (95% CI, 2.81-30.28)	
**(16)**	Spain, Madrid	Cohort study	Children who were part of a longitudinal study on obesity	1,985	May 2021	RT-PCR and RAT	Symptomatic or close contact testing	Ancestral/Alpha	General and abdominal obesity	RR	Children with general obesity: RR 2.53 (95% CI, 1.56-4.10)<br><br>Abdominal obesity: RR 2.56 (95% CI, 1.55-4.21)	

Study ref	Country, nation/ city	Study design	Study population	Number of participants	Study time frame	Lab method for SARS-CoV-2 confirmation	Testing criteria	Variant(s)/ variant subgroup(s)	Measured risk factor(s)/ exposure(s)	Measure of association	Factors positively associated with SARS-CoV-2 infection	Factors negatively associated with SARS-CoV-2 infection
**(17)**	United Kingdom, England	Cohort study	Adults aged 18 years and older who were registered with a primary care practice on Feb 1, 2020	17,288,532	Feb 2020 - Aug 2020	RT-PCR	PCR swabs for symptomatic individuals in UK	Ancestral/Alpha	Ethnicity	HR	Ethnicity:<br>White (ref)<br>South Asian: aHR 1·99 (95% CI, 1·94–2·04)<br>Black: aHR 1·69<br>(95% CI, 1·62–1·77)<br>Mixed: aHR 1·49<br>(95% CI, 1·39–1·59)<br>Other: aHR 1·20 (<br>95% CI, 1·14–1·28)	
**(18)**	United Kingdom	Case control	Users of the COVID Symptom app in the UK and reported vaccination doses	1,240,009	Dec 2020 - Jul 2021	RT-PCR or RAT	No criteria to prompt testing, people without recent test results were excluded	Ancestral/Alpha	Frailty, index of multiple deprivation, comorbidities	OR	Frailty: OR 1.93 (95% CI, 1.50-2.48)<br><br>Increased deprivation (low on the IMD of 1-3): OR 1.11 (95% CI, 1.01-1.23)<br><br>Kidney disease: aOR 1.95 (95% CI, 1.14-3.31)<br>Heart disease: aOR 1.30 (95% CI, 1.03-1.65)<br>Lung disease: aOR 1.27 (95% CI, 1.02-1.59)	
**(19)**	United Kingdom	Cohort study	Individuals enrolled in the UK biobank, and tested in England	4,509	Mar 2020 - May 2020	RT-PCR	Symptomatic individuals or health care workers	Ancestral	Age, gender, ethnicity, household size, obesity, income, education level.	OR	Male: aOR 1.10 (95% CI, 1.06-1.14)<br><br>Ethnicity:<br>White: (ref)<br>Black ethnicity: aOR 1.05 (95% CI, 1.02-1.08)<br><br>Lower education level: aOR 1.09 (95% CI, 1.05-1.13)<br><br>Increased household size: aOR 1.05 (95% CI, 1.02-1.08)<br><br>Income <18,000 GBP: aOR 1.06<br>(95% CI, 1.01-1.10)<br><br>Severely obese vs non-overweight:<br>aOR 1.05 (95% CI, 1.02-1.08)	

Study ref	Country, nation/ city	Study design	Study population	Number of participants	Study time frame	Lab method for SARS-CoV-2 confirmation	Testing criteria	Variant(s)/ variant subgroup(s)	Measured risk factor(s)/ exposure(s)	Measure of association	Factors positively associated with SARS-CoV-2 infection	Factors negatively associated with SARS-CoV-2 infection
**(20)**	United Kingdom	Cohort study	Individuals enrolled in the Biobank grouped as with or without mental disorders	77,217	Dec 2019 - Jul 2021	RT-PCR	Screening all symptomatic and asymptomatic patients	Ancestral/Alpha/Delta	Ethnicity, Townsend deprivation, comorbidities, BMI, education level, fair or poor health level, mental disorders/illness	OR	Mental illness: OR 1.11 (95% CI, 1.04-1.18)<br><br>Low education level with mental disorders (MD): OR 1.50 (95% CI, 1.30-1.72)<br>Low education level without MD: OR 1.56 (95% CI, 1.49-1.63)<br><br>Non-white ethnicity: OR 1.31 (95% CI, 1.06-1.63)<br><br>Respiratory disease: with MD: OR 1.14 (95% CI, 1.02-1.27)<br>Without MD: OR 1.25 (95%CI, 1.19-1.32)<br>Cerebrovascular disease: With MD: OR 1.74 (95% CI, 1.45–2.10)<br>Without MD: OR 1.19 (95% CI, 1.02–1.38) Diabetes With MD: OR 1.18 (95% CI, 1.00-1.39)<br>Without MD: OR 1.35 (95% CI, 1.24-1.47) Hypertension: With MD: OR 1.13 (95% CI, 1.00–1.28)<br>Without MD: OR 1.13 (95% CI, 1.07-1.20)<br>Male sex: OR 1.07 (95% CI, 1.03-1.11), Low Townsend score: OR 1.03 (95% CI.1.03-1.04),<br>Fair or poor health rating: OR 1.09 (95% CI, 1.03-1.14)<br>Smoking: OR 1.12 (95% CI, 1.08-1.17) High physical activity OR 1.10 (95% CI, 1.05-1.15)<br>Obesity: OR 1.20 (95% CI, 1.15-1.25)	

Study ref	Country, nation/ city	Study design	Study population	Number of participants	Study time frame	Lab method for SARS-CoV-2 confirmation	Testing criteria	Variant(s)/ variant subgroup(s)	Measured risk factor(s)/ exposure(s)	Measure of association	Factors positively associated with SARS-CoV-2 infection	Factors negatively associated with SARS-CoV-2 infection
**(21)**	United Kingdom	Cross-sectional study	Patients in the Oxford RCGP research and surveillance centre network	3,802	Jan 2020 - Apr 2020	RT-PCR	Symptomatic individuals or health care workers	Ancestral	Age, sex, ethnicity, index of multiple deprivation quintiles, obesity, residential rurality	OR	Male Sex: aOR 1.55: (95% CI, 1.27-1.89)<br><br>Age:<br>0-17: (ref)<br>18-39: 2.83 (95% CI, 1.69-4.74)<br>40–64: aOR 5.36 (95% CI, 3.28-8.76)<br>65-74: aOR 4.41 (95% CI, 2.52-7.69)<br>75 years or older: aOR 5.23 (95% CI, 3.00-9.09)<br><br>Ethnicity: White: (ref)<br>Black ethnicity: aOR 4.75 (95% CI, 2.65-8.51)<br><br>Urban residential status: aOR 4.59 (95% CI, 3.57-5.90)<br><br>Socioeconomic deprivation level:<br>5 (least deprived); (ref)<br>4: aOR 1.51 (95% CI,1.13-2.03)<br>3: aOR 2.35 (95% CI,1.78-3.11)<br>1 and 2 (most deprived): aOR 2.03 (95% CI, 1.51-2.71)<br><br>BMI:<br>Normal weight: (ref)<br>Obese: aOR 1.26 (95% CI, 1.04-1.91)	

Study ref	Country, nation/ city	Study design	Study population	Number of participants	Study time frame	Lab method for SARS-CoV-2 confirmation	Testing criteria	Variant(s)/ variant subgroup(s)	Measured risk factor(s)/ exposure(s)	Measure of association	Factors positively associated with SARS-CoV-2 infection	Factors negatively associated with SARS-CoV-2 infection
**(22)**	United Kingdom	Cohort study	Middle aged individuals enrolled in the UK biobank	18,221	Mar 2020 - Sep 2020	RT-PCR	Symptomatic individuals or health care workers	Ancestral	Gender, shift work type, ethnicity, Townsend measure of deprivation, household income	OR/aOR	Male gender: aOR 1.22 (95% CI, 1.03-1.45)<br><br>Night shift employees: aOR 1.85 (95% CI, 1.42-2.41)<br><br>Ethnic minorities:<br>aOR 1.87 (95%CI, 1.41-2.48)<br><br>Townsend score:<br>Q1 (least deprived: (ref)<br>Q4: OR 1.45 (95% CI, 1.18-1.80)<br>Q5 (most deprived): OR 1.73 (95% CI, 1.29-2.33)	Annual household income: >52,000 GBP: OR 0.78 (95% CI, 0.64-0.95)
**(23)**	United Kingdom	Cohort study	Individuals enrolled in the UK biobank, and tested in England or Scotland	447,296	Jan 2020 - Feb 2021	RT-PCR	Not specified	Alpha	Psychiatric diagnosis	OR	Major depressive disorder (MDD):<br>aOR 1.24 (95% CI, 1.18-1.30)	
**(24)**	United Kingdom	Cohort study	Individuals diagnosed with cancer after enrolling into the UK biobank	18,917	Mar 2020 - Aug 2020	RT-PCR	Symptomatic testing at health care service	Ancestral	Townsend deprivation index, ethnicity, employment status	RR	Lowest Townsend quintile:<br>aRR 2.52 (95% CI, 1.00-6.33)<br><br>Black patients compared to white patients:<br>aRR 5.79 (95% CI, 1.88-17.85)<br><br>Unemployed: aRR 1.41 (95% CI, 1.20-1.67)	

Study ref	Country, nation/ city	Study design	Study population	Number of participants	Study time frame	Lab method for SARS-CoV-2 confirmation	Testing criteria	Variant(s)/ variant subgroup(s)	Measured risk factor(s)/ exposure(s)	Measure of association	Factors positively associated with SARS-CoV-2 infection	Factors negatively associated with SARS-CoV-2 infection
**(25)**	United Kingdom	Cohort study	UK Biobank participants	428,199	Mar 2020 - May 2020	RT-PCR	Patients in hospital (emergency department and inpatient) settings	Ancestral	Long term health conditions, number of medications, age, ethnicity, Townsend quintile	RR	Multimorbidity (2 or more long term conditions): RR 1.48 (95% CI, 1.28-1.71)<br><br>Non-white ethnicities with multimorbidities: RR 2.81 (95% CI, 2.09-3.78)<br><br>Age >65 years with multimorbidities: RR 1.29 (95% CI, 1.09-1.53)<br><br>Most deprived on the Townsend quintile scale with multimorbidities: OR 2.79 (95% CI, 2.00-3.90)<br><br>BMI >40 with multimorbidities: RR 2.66 (95% CI, 1.88-3.76)	
**(26)**	United Kingdom	Prospective cohort	People who attended UK Biobank baseline assessment centres in England	2,658	Mar 2020 - May 2020	RT-PCR	Not specified	Ancestral	Ethnicity, education level, socioeconomic deprivation	RR	Ethnicity:<br>White British: (ref)<br>Black ethnicity: aRR 1.49 (95% CI, 1.215-1.825)<br>South Asian: aRR 1.490 (95% CI, 1.189-1.868)<br><br>Socioeconomic deprivation:<br>Most deprived (Q4): aRR 1.209 (95% CI, 1.038-1.408)<br><br>Lowest education level: aRR 1.227 (95% CI, 1.055-1.428)	
**(27)**	United Kingdom	Cohort study	UK Biobank participants	402,978	Feb 2020 - Jun 2020	RT-PCR	Not specified	Ancestral	Smoking	IRR	Current smoking: aIRR 1.27 (95% CI, 1.08-1.50)<br>Previous smoking: aIRR 1.26 (95% CI, 1.13-1.40)	

Study ref	Country, nation/ city	Study design	Study population	Number of participants	Study time frame	Lab method for SARS-CoV-2 confirmation	Testing criteria	Variant(s)/ variant subgroup(s)	Measured risk factor(s)/ exposure(s)	Measure of association	Factors positively associated with SARS-CoV-2 infection	Factors negatively associated with SARS-CoV-2 infection
**(28)**	United Kingdom	Case control	UK Biobank participants	66,732	Mar 2020 - Mar 2021	RT-PCR	Symptomatic presentation to hospital	Ancestral/Alpha	Index of multiple deprivation (IMD) - equivalent of SES, air pollution, proximity to green/natural spaces	OR	Lower Index of Multiple Deprivation (IMD) score: OR 1.207 (95% CI, 1.18-1.23)<br><br>Air pollution:<br>Nitrogen oxide levels: OR 1.067 (95% CI, 1.05-1.09)<br><br>Particulate matter: OR 1.063 (95% CI, 1.04-1.09)<br><br>Nitrogen dioxide levels (2005): OR 1.048 (95% CI, 1.02-1.07)	Proximity to green space:<br>300m: OR 0.925 (95% CI, 0.906-0.944)<br>1000m: OR 0.940 (95% CI, 0.920-0.960)<br><br>Proximity to natural environment:<br>300m: OR 0.939 (95% CI, 0.920-0.960)<br>1000m: OR 0.941 (95% CI, 0.921-0.961)
**(29)**	United States of America	Cross-sectional study	Adults who were tested or admitted at Sharp Grossman hospital, Southern California	25,216	Mar 2020 - Jan 2021	RT-PCR	Symptomatic testing at health care service	Ancestral	Race/ethnicity	OR	Arab Americans compared to Non-Hispanic White: aOR 3.83 (95% CI, 3.29-4.46)<br><br>Arab Americans compared to Black ethnicity: aOR 2.34 (95% CI, 1.91-2.88)	
**(30)**	United States of America	Cross-sectional study	Adults who were tested or admitted at Sharp Grossman hospital, Southern California	2,744	Mar 2020 - Jan 2021	RT-PCR	Symptomatic testing at health care service	Ancestral	Residential zip code/poverty level, gender, age	OR	Living in a zip code in which >12% of families live in poverty: aOR 1.25 (95% CI, 1.04-1.51)	Gender:<br>Female: aOR 0.77 (95% CI, 0.64-0.92)<br><br>Age:<br>18-29: 0.36 (95% CI, 0.21-0.60)<br>>70: (ref)
**(31)**	United States of America	Cross-sectional study	People getting tested at testing sites in Los Angeles	2,141,127	Jun 2020 - Dec 2020	RT-PCR	Drive through testing in LA, symptomatic testing	Ancestral	Race/ethnicity, annual household income, % with health insurance and % employed in testers zip code	OR	Race/ethnicity:<br>Hispanic: (ref)<br>White: aOR 4.19 (95% CI, 4.13-4.26)<br>Asian: aOR 4.05 (95% CI, 3.97-4.13)<br>Black: aOR 2.84 (95% CI, 2.77-2.93)<br>Other/unknown: aOR 2.50 (95% CI, 2.42-2.58)	

Study ref	Country, nation/ city	Study design	Study population	Number of participants	Study time frame	Lab method for SARS-CoV-2 confirmation	Testing criteria	Variant(s)/ variant subgroup(s)	Measured risk factor(s)/ exposure(s)	Measure of association	Factors positively associated with SARS-CoV-2 infection	Factors negatively associated with SARS-CoV-2 infection
											Multiracial: aOR 4.24 (95% CI, 4.05-4.44)<br><br>Annual average income >$110,000: (ref)<br>Zip codes with an average annual household income of less than 65,000: aOR 1.77 (95% CI, 1.72-1.82)<br>Annual incomes of $65,000 to $84,999 aOR 1.62 (95% CI, 1.58-1.66)<br><br>Zip codes with less than 85% who have healthcare coverage aOR 1.29 (95% CI, 1.25-1.33)<br><br>Zip codes with less than 60% over 16 employed aOR 1.42 (95% CI, 1.41-1.44)	
**(32)**	United States of America	Retrospective cohort study	Patients in Providence health services tested in California, Oregon, and Washington.	629,953	Mar 2020 - Dec 2020	RT-PCR	Symptomatic presentation to hospital	Ancestral	Ethnicity, gender, age, % limited English, Overweight/Obese, income level	OR	Ethnicity:<br>White: (ref)<br>Hispanic: aOR3.09 (95% CI, 2.99-3.18)<br>Native Hawaiian/Pacific Islanders aOR 2.23 (95% CI, 2.01-2.48) Black: aOR 1.35; (95% CI, 1.28-1.43)<br>Asian: aOR 1.31 (95% CI, 1.25-1.38)<br><br>Male gender: aOR, 1.27 (95% CI, 1.25-1.29)<br><br>Age (per year increase): aOR 1.12 (95% CI, 1.1-1.14)<br><br>% Limited English: aOR 1.13 (95% CI, 1.11-1.15)<br><br>Overweight: aOR 1.15 (95% CI, 1.12-1.19)<br>Obesity class 1: aOR 1.21 (95% CI, 1.16-1.25)	Median income (log): aOR 0.84 (95% CI, 0.83-0.85)

Study ref	Country, nation/ city	Study design	Study population	Number of participants	Study time frame	Lab method for SARS-CoV-2 confirmation	Testing criteria	Variant(s)/ variant subgroup(s)	Measured risk factor(s)/ exposure(s)	Measure of association	Factors positively associated with SARS-CoV-2 infection	Factors negatively associated with SARS-CoV-2 infection
**(33)**	United States of America	Cohort study	Non-hospitalised patients under 21 years old	1,796	Mar 2020 - Jun 2020	RT-PCR	Symptomatic presentation to hospital	Ancestral	Age, poverty level of residential county, ethnicity	OR	> 10 years old: OR 2.19 (95% CI, 1.5-3.2)<br><br>From counties with more than 15% of residents under 18 living below the poverty line: OR 1.5 (95% CI, 1.06-2.1)<br>Hispanic/Latino: OR 3 (95% CI, 2.29-4.03)	
**(34)**	United States of America	Cross-sectional study	All aged individuals in Oakland, California	1,034	Sep 2020 - Sep 2020	RT-PCR	Free testing to any individual in the area.	Ancestral	Gender and ethnicity	OR	Mayan: aOR 16.66 (95% CI, 3.54-78.41)<br>non-Mayan Latinx: aOR 8.48 (95% CI, 1.91-37.67)<br><br>Male gender:<br>aOR 2.25 (95% CI, 1.16-4.39)	
**(35)**	United States of America	Cross-sectional study	Veterans' Health Administration members	88,747	Feb 2020 - May 2020	RT-PCR	Not specified, but mentioned some likely to be due to routine screening testing	Ancestral	Gender, race, ethnicity, age, obesity, rurality, smoking status	OR	Male: aOR 1.45 (95% CI, 1.34-1.57)<br><br>Age:<br>18-49: 1 (ref)<br>50-64: aOR 1.18 (95% CI, 1.10-1.27)<br>65-79: aOR 1.46 (95% CI, 1.35-1.57)<br>>80 years old: aOR, 2.16 (95% CI, 1.97-2.37)<br><br>Race:<br>Black: aOR 2.15 (95% CI, 2.05-2.26)<br>American Indian/Alaskan Native/Native Hawaiian/Pacific Islander: aOR 1.26 (95% CI, 1.05-1.52)<br><br>Ethnicity:<br>Non-Hispanic: 1 (ref)<br>Hispanic: aOR 1.52 (95% CI, 1.40-1.65)<br><br>Rural setting: 1(ref)<br>Urban setting: 1.78 (95% CI, 1.70-1.87)	Smoker:<br>Never: 1 (ref)<br>Former: aOR 0.92 (95% CI, 0.88-0.97)<br>Current: aOR 0.52 (95% CI, 0.49-0.57)<br>Unknown: aOR 0.84 (95% CI, 0.78-0.91)

Study ref	Country, nation/ city	Study design	Study population	Number of participants	Study time frame	Lab method for SARS-CoV-2 confirmation	Testing criteria	Variant(s)/ variant subgroup(s)	Measured risk factor(s)/ exposure(s)	Measure of association	Factors positively associated with SARS-CoV-2 infection	Factors negatively associated with SARS-CoV-2 infection
											Overweight: aOR 1.16 (95% CI, 1.09-1.24)<br>Class I Obesity: aOR 1.32 (95% CI, 1.23-1.41)<br>Class II/III obesity: aOR 1.44 (95% CI, 1.34-1.56)	
**(36)**	United States of America	Retrospective cohort study	In and out-patients at the University of Miami Hospital and Clinics	15,473	Mar 2020 - Jul 2020	RT-PCR	Symptomatic presentation to hospital	Ancestral	Ethnicity/race	OR	Non-Hispanic black: aOR 2.55 (95% CI, 2.05-3.17)<br><br>Hispanic white:<br>aOR 2.04 (95% CI, 1.69-2.46)<br><br>Hispanic Black:<br>aOR 2.25 (95% CI, 1.46-3.38).	
**(37)**	United States of America	Cohort Study	Enrolled Veteran Affairs members in United States	9,127,673	Feb 2020 - Mar 2021	RT-PCR	Symptomatic presentation to VA hospital	Ancestral/Alpha	Place of residence (rurality), race/ethnicity, BMI, age, comorbidities	OR	Black race: aOR 1.39 (95% CI, 1.37-1.41)<br><br>Hispanic ethnicity: aOR 1.64 (95% CI, 1.62-1.67)<br><br>Urban residence:<br>aOR 2.02 (95% CI, 1.83-2.22)<br><br>BMI:<br>30: aOR 1.23 (95% CI, 1.21-1.25)<br>35: aOR 1.38 (95% CI, 1.36-1.39)<br>40: aOR 1.51 (95% CI, 1.49-1.53)<br><br>Charlson comorbidity Index:<br>0 to 1: 1(ref)<br>2 to 3: aOR 2.16 (95% CI, 2.14-2.19)<br>4 to 5: aOR 2.56 (95% CI, 2.52-2.60)<br>>6: aOR 3.16 (95% CI, 3.12-3.21)	Age (years):<br>25: aOR 0.77 (95% CI, 0.76-0.79)<br>35: aOR 0.92 (95% CI, 0.91-0.92)<br>45: 1 (ref)<br>55: aOR 0.89 (95% CI, 0.88-0.89)<br>65: aOR 0.71 (95% CI, 0.70-0.73)<br>75: aOR 0.64 (95% CI, 0.63-0.65)<br>85: aOR 0.58 (95% CI, 0.57-0.59)

Study ref	Country, nation/ city	Study design	Study population	Number of participants	Study time frame	Lab method for SARS-CoV-2 confirmation	Testing criteria	Variant(s)/ variant subgroup(s)	Measured risk factor(s)/ exposure(s)	Measure of association	Factors positively associated with SARS-CoV-2 infection	Factors negatively associated with SARS-CoV-2 infection
**(38)**	United States of America	Cohort study	Patients in a family medical centre in Maryland	1,781	Mar 2020 - Jun 2020	RT-PCR	Symptomatic testing at health care service	Ancestral	Race/ethnicity, socioeconomic deprivation	OR	Black non-Hispanic: OR 1.75 (95% CI, 1.18-2.59)<br>Hispanic patients: OR 5.40 (95% CI, 3.11-9.38)<br>Higher socioeconomic deprivation: OR 1.68 (95% CI, 1.25-2.28)	
**(39)**	United States of America	Test-negative case-control	Children (0-18y) treated in Rush-affiliated outpatient clinics, emergency departments and inpatient admissions in Chicago	8,462	Mar 2020 - Dec 2020	RT-PCR	Initially included having severe disease, being in a risk group or being health personnel; this later changed to include everyone with symptoms (e.g., cough or fever) or having been in close contact	Ancestral	Ethnicity, age group, median family income, Obesity, neighbourhood ethnic minority proportion	OR	Hispanic/Latinx: OR 2.45 (95% CI, 1.99-3.03)<br>Black/African American OR 1.31 (95% CI, 1.03-1.66)<br><br>Median family incomes of < $50,000:<br>OR 1.36 (95% CI, 1.17-1.60)<br><br>Overweight/obese: OR 1.27 (95% CI, 1.02-1.58)<br><br>Children aged 10-14 OR 1.70 (95% CI, 1.39-2.08)<br>Aged 15-18 OR 2.06 (95% CI, 1.71-2.47)<br><br>Neighbourhood with greater than 50% minority populations: OR 1.45 (95% CI, 1.17-1.80).	
**(40)**	United States of America	Cross-sectional study	Outpatients from a series of health clinics in Wisconsin	2,595	Mar 2020 - Mar 2020	RT-PCR	All patients presenting in a health service	Ancestral	Patient race, age, sex	OR	Black race: OR 5.37 (95% CI, 3.94-7.29)<br>Males: OR 1.55 (95% CI, 1.21-2.00)<br><br>>60 years old OR 2.04 (95% CI, 1.53-2.73)	

Study ref	Country, nation/ city	Study design	Study population	Number of participants	Study time frame	Lab method for SARS-CoV-2 confirmation	Testing criteria	Variant(s)/ variant subgroup(s)	Measured risk factor(s)/ exposure(s)	Measure of association	Factors positively associated with SARS-CoV-2 infection	Factors negatively associated with SARS-CoV-2 infection
**(41)**	United States of America	Cohort study	Patients in Providence health services throughout Alaska, Washington, Oregon, Montana, and California	34,503	Feb 2020 - Apr 2020	RT-PCR	Symptomatic testing at health care service	Ancestral	Age, gender, housing and transportation security, race, ethnicity, non-English language, comorbidities, neighbourhood financial insecurity, employment status, education level, and aged care residences	OR	Age:<br><18: aOR 0.33 (95% CI, 0.22-0.49)<br>18-29: 1 (ref)<br>40-49: aOR 1.27 (95% CI, 1.06-1.52)<br>50-59: aOR 1.69 (95% CI, 1.41-2.02)<br>60-69: aOR 1.65 (95% CI, 1.36-2.01)<br>70-79: aOR 1.59 (95% CI, 1.24-2.05)<br>80+: aOR 1.64 (95% CI, 1.24-2.17)<br><br>Male gender: OR 1.32 (95% CI, 1.21-1.44)<br><br>Asian race: OR 1.43 (95% CI, 1.18-1.72)<br>Black/African American race: OR 1.51 (95% CI, 1.25-1.83)<br><br>Hispanic or Latino ethnicity: OR 2.07 (95% CI, 1.77-2.41)<br><br>Language spoken:<br>Sino-Tibetan: aOR 1.98 (95% CI, 1.38-2.84)<br>Spanish: aOR 1.60 (95% CI, 1.31-1.94)<br>Non-English language speaking: OR 2.09 (95% CI, 1.7-2.57)<br><br>Neighbourhood with financial insecurity: OR 1.10 (95% CI, 1.01-1.25)<br><br>Housing insecurity: OR 1.32 (95% CI, 1.16-1.5)<br>Transportation insecurity: OR 1.11 (95% CI, 1.02-1.23)<br><br>Senior living community OR 1.69 (95% CI, 1.23-2.32)	

Study ref	Country, nation/ city	Study design	Study population	Number of participants	Study time frame	Lab method for SARS-CoV-2 confirmation	Testing criteria	Variant(s)/ variant subgroup(s)	Measured risk factor(s)/ exposure(s)	Measure of association	Factors positively associated with SARS-CoV-2 infection	Factors negatively associated with SARS-CoV-2 infection
											Diabetes OR 1.40 (95% CI, 1.22-1.61)<br>Severely obese OR 1.58 (95% CI, 1.31-1.91)<br><br>Education/Employment:<br>Student: 1 (ref)<br>Education < 12 years: aOR 1.32 (95% CI, 1.01-1.14)<br>Employed: aOR 1.85 (95% CI, 1.39-2.46)<br>Not employed: aOR 1.41 (95% CI, 1.05-1.91)<br>Retired: aOR 2.06 (95% CI, 1.54-2.76)	
**(42)**	United States of America	Retrospective cohort study	Pregnant women presenting with potential COVID-19 concern	1,418	Mar 2020 - May 2020	RT-PCR	Screening all symptomatic and asymptomatic patients	Ancestral	Have any living children (1-4+)	OR	Having one living child: OR 2.5 (95% CI, 1.5-4.0)<br>Having two living children: OR 2.1 (95% CI, 1.0-4.1)<br>Having three living children: OR 4.1 (95% CI, 1.6-10.5)<br>Having at least 4 living children: OR 7.0 (95% CI, 2.8-17.7)	
**(43)**	United States of America	Cross-sectional study	Recent close contacts in Houston and return travellers from hotspots	20,228	Mar 2020 - May 2020	RT-PCR	Symptomatic, close contact or returned traveller testing	Ancestral	Ethnicity/race, gender, age, diabetes	OR	Ethnicity:<br>Non-Hispanic Black (vs Non-Hispanic White): aOR 2.23 (95% CI, 1.90-2.60)<br>Hispanic (vs non-Hispanic): aOR 1.95 (95% CI, 1.72-2.20)<br><br>*Non-Hispanic Black vs non-Hispanic White:*<br><br>Male: aOR 1.20 (95% CI, 1.04-1.39)<br><br>Age:<br>Up to 35: 1 (ref)<br>36-50: aOR 1.35 (95% CI, 1.07-1.72)<br>51-75: aOR 1.60 (95% CI, 1.21-2.11)<br>>75: aOR 2.20 (95% CI, 1.52-3.19)	

Study ref	Country, nation/ city	Study design	Study population	Number of participants	Study time frame	Lab method for SARS-CoV-2 confirmation	Testing criteria	Variant(s)/ variant subgroup(s)	Measured risk factor(s)/ exposure(s)	Measure of association	Factors positively associated with SARS-CoV-2 infection	Factors negatively associated with SARS-CoV-2 infection
											Diabetes: aOR 1.42 (95% CI, 1.17-1.71)<br><br>*Hispanic vs non-Hispanic:*<br><br>Male: aOR 1.17 (95% CI, 1.05-1.32)<br><br>Age:<br>Up to 35: 1 (ref)<br>36-50: aOR 1.42 (95% CI, 1.20-1.68)<br>51-75: aOR 1.71 (95% CI, 1.39-2.11)<br>>75: 2.08 (95% CI, 1.56-2.77)<br><br>Diabetes: aOR 1.62 (95% CI, 1.40-1.87)	

aIRR = adjusted incidence risk ratio; aOR = adjusted odds ratio; aRR = adjusted risk ratio; BMI = body mass index; HR = hazard ratio; IRR = incidence risk ratio; OR = odds ratio; PR = prevalence ratio; RAT = rapid antigen test; RR = risk ratio; RT-PCR = reverse transcription-polymerase chain reaction; SES = socioeconomic status; VA = Veteran's Affairs

References

1. Sebastiao CS, Neto Z, Martinez P, Jandondo D, Antonio J, Galangue M, et al. Sociodemographic characteristics and risk factors related to SARS-CoV-2 infection in Luanda, Angola. PloS one. 2021;16(3):e0249249.

2. Varela AR, Florez LJH, Tamayo-Cabeza G, Contreras-Arrieta S, Restrepo SR, Laajaj R, et al. Factors Associated With SARS-CoV-2 Infection in Bogota, Colombia: Results From a Large Epidemiological Surveillance Study. Lancet Regional Health Americas. 2021;2(9918232503006676):100048.

3. Galmiche S, Charmet T, Schaeffer L, Paireau J, Grant R, Chény O, et al. Exposures associated with SARS-CoV-2 infection in France: A nationwide online case-control study. Lancet Reg Health Eur. 2021;7:100148.

4. Grant R, Charmet T, Schaeffer L, Galmiche S, Madec Y, Von Platen C, et al. Impact of SARS-CoV-2 Delta variant on incubation, transmission settings and vaccine effectiveness: Results from a nationwide case-control study in France. Lancet Reg Health Eur. 2022;13:100278.

5. Bella A, Akbar MT, Kusnadi G, Herlinda O, Regita PA, Kusuma D. Socioeconomic and Behavioral Correlates of COVID-19 Infections among Hospital Workers in the Greater Jakarta Area, Indonesia: A Cross-Sectional Study. International Journal of Environmental Research and Public Health. 2021;18(10).

6. D'Ambrosi F, Iurlaro E, Tassis B, Di Maso M, Erra R, Cetera GE, et al. Sociodemographic characteristics of pregnant women tested positive for COVID-19 admitted to a referral center in Northern Italy during lockdown period. The Journal of Obstetrics and Gynaecology Research. 2021;47(5):1751-6.

7. Magnusson K, Nygard K, Methi F, Vold L, Telle K. Occupational risk of COVID-19 in the first versus second epidemic wave in Norway, 2020. Euro surveillance : bulletin Europeen sur les maladies transmissibles = European communicable disease bulletin. 2021;26(40).

8. Stordal K, Ruiz PL-D, Greve-Isdahl M, Suren P, Knudsen PK, Gulseth HL, et al. Risk factors for SARS-CoV-2 infection and hospitalisation in children and adolescents in Norway: a nationwide population-based study. BMJ open. 2022;12(3):e056549.

9. Magalhaes JPM, Ribeiro AI, Caetano CP, Sa Machado R. Community socioeconomic deprivation and SARS-CoV-2 infection risk: findings from Portugal. European Journal of Public Health. 2022;32(1):145-50.

10. Leite A, Leao T, Soares P, Severo M, Moniz M, Lucas R, et al. A Case-Control Study of Contextual Factors for SARS-CoV-2 Transmission. Frontiers in public health. 2021;9(101616579):772782.

11. Chun S-Y, Kim DW, Lee SA, Lee SJ, Chang JH, Choi YJ, et al. Does Diabetes Increase the Risk of Contracting COVID-19? A Population-Based Study in Korea. Diabetes & metabolism journal. 2020;44(6):897-907.

12. Oh TK, Choi J-W, Song I-A. Socioeconomic disparity and the risk of contracting COVID-19 in South Korea: an NHIS-COVID-19 database cohort study. BMC public health. 2021;21(1):144.

13. Aguilar-Palacio I, Maldonado L, Malo S, Sanchez-Recio R, Marcos-Campos I, Magallon-Botaya R, et al. COVID-19 Inequalities: Individual and Area Socioeconomic Factors (Aragon, Spain). International journal of environmental research and public health. 2021;18(12).

14. Castilla J, Guevara M, Miqueleiz A, Baigorria F, Ibero-Esparza C, Navascues A, et al. Risk Factors of Infection, Hospitalization and Death from SARS-CoV-2: A Population-Based Cohort Study. Journal of clinical medicine. 2021;10(12).

15. Morante-Garcia W, Zapata-Boluda RM, Garcia-Gonzalez J, Campuzano-Cuadrado P, Calvillo C, Alarcon-Rodriguez R. Influence of Social Determinants of Health on COVID-19 Infection in Socially Vulnerable Groups. International journal of environmental research and public health. 2022;19(3).

16. Ortiz-Pinto MA, de Miguel-Garcia S, Ortiz-Marron H, Ortega-Torres A, Cabanas G, Gutierrez-Torres LF, et al. Childhood obesity and risk of SARS-CoV-2 infection. International journal of obesity (2005). 2022(101256108).

17. Mathur R, Rentsch CT, Morton CE, Hulme WJ, Schultze A, MacKenna B, et al. Ethnic differences in SARS-CoV-2 infection and COVID-19-related hospitalisation, intensive care unit admission, and death in 17 million adults in England: an observational cohort study using the OpenSAFELY platform. Lancet (London, England). 2021;397(10286):1711-24.

18. Antonelli M, Penfold RS, Merino J, Sudre CH, Molteni E, Berry S, et al. Risk factors and disease profile of post-vaccination SARS-CoV-2 infection in UK users of the COVID Symptom Study app: a prospective, community-based, nested, case-control study. The Lancet Infectious diseases. 2022;22(1):43-55.

19. Chadeau-Hyam M, Bodinier B, Elliott J, Whitaker MD, Tzoulaki I, Vermeulen R, et al. Risk factors for positive and negative COVID-19 tests: a cautious and in-depth analysis of UK biobank data. International journal of epidemiology. 2020;49(5):1454-67.

20. Dai XJ, Shao Y, Ren L, Tao W, Wang Y. Risk factors of COVID-19 in subjects with and without mental disorders. Journal of Affective Disorders. 2022;297((Dai) School of Chemical Biology and Biotechnology, Peking University Shenzhen Graduate School, Shenzhen 518020, China(Dai, Shao, Ren, Tao, Wang) Shenzhen Mental Health Center, Shenzhen Kangning Hospital, 1080#, Cuizhu Rd, Luohu District, Shenzhen 518003):102-11.

21. de Lusignan S, Dorward J, Correa A, Jones N, Akinyemi O, Amirthalingam G, et al. Risk factors for SARS-CoV-2 among patients in the Oxford Royal College of General Practitioners Research and Surveillance Centre primary care network: a cross-sectional study. The Lancet Infectious diseases. 2020;20(9):1034-42.

22. Fatima Y, Bucks RS, Mamun AA, Skinner I, Rosenzweig I, Leschziner G, et al. Shift work is associated with increased risk of COVID-19: Findings from the UK Biobank cohort. Journal of sleep research. 2021;30(5):e13326.

23. Hassan L, Peek N, Lovell K, Carvalho AF, Solmi M, Stubbs B, et al. Disparities in COVID-19 infection, hospitalisation and death in people with schizophrenia, bipolar disorder, and major depressive disorder: a cohort study of the UK Biobank. Molecular psychiatry. 2022;27(2):1248-55.

24. Lee SF, Niksic M, Rachet B, Sanchez M-J, Luque-Fernandez MA. Socioeconomic Inequalities and Ethnicity Are Associated with a Positive COVID-19 Test among Cancer Patients in the UK Biobank Cohort. Cancers. 2021;13(7).

25. McQueenie R, Foster HME, Jani BD, Katikireddi SV, Sattar N, Pell JP, et al. Multimorbidity, polypharmacy, and COVID-19 infection within the UK Biobank cohort. PloS one. 2020;15(8):e0238091.

26. Niedzwiedz CL, O'Donnell CA, Jani BD, Demou E, Ho FK, Celis-Morales C, et al. Ethnic and socioeconomic differences in SARS-CoV-2 infection: prospective cohort study using UK Biobank. BMC medicine. 2020;18(1):160.

27. Prats-Uribe A, Xie J, Prieto-Alhambra D, Petersen I. Smoking and COVID-19 Infection and Related Mortality: A Prospective Cohort Analysis of UK Biobank Data. Clinical epidemiology. 2021;13(101531700):357-65.

28. Scalsky RJ, Chen Y-J, Ying Z, Perry JA, Hong CC. The Social and Natural Environment's Impact on SARS-CoV-2 Infections in the UK Biobank. International journal of environmental research and public health. 2022;19(1).

29. Abuelezam NN, Greenwood KL, Galea S, Al-Naser R. Differential COVID-19 testing, admissions, and mortality for Arab Americans in Southern California. PloS one. 2022;17(4):e0267116.

30. Abuelezam NN, Greenwood KL, Al-Ani M, Galea S, Al-Naser R. Risk Factors for COVID-19 Positivity and Hospital Admission Among Arab American Adults in Southern California. Public health reports (Washington, DC : 1974). 2022((Abuelezam) William F. Connell School of Nursing, Boston College, MA, Chestnut Hill, United States(Greenwood) Sharp HealthCare Center for Research, San Diego, CA, USA(Al-Ani) UC San Diego Health, University of California at San Diego, San Diego, CA, USA(G):333549221083740.

31. Allan-Blitz L-T, Goldbeck C, Hertlein F, Turner I, Klausner JD. Association of Lower Socioeconomic Status and SARS-CoV-2 Positivity in Los Angeles, California. Journal of preventive medicine and public health = Yebang Uihakhoe chi. 2021;54(3):161-5.

32. Dai CL, Kornilov SA, Roper RT, Cohen-Cline H, Jade K, Smith B, et al. Characteristics and Factors Associated With Coronavirus Disease 2019 Infection, Hospitalization, and Mortality Across Race and Ethnicity. Clinical infectious diseases : an official publication of the Infectious Diseases Society of America. 2021;73(12):2193-204.

33. Denny V, Shah N, Petro K, Choksey K, DeSantis E, Hintz M, et al. Impact of outpatient SARS-CoV-2 infections in minority children. Medicine. 2021;100(8):e24895.

34. Esaryk EE, Wesson P, Fields J, Rios-Fetchko F, Lindan C, Bern C, et al. Variation in SARS-CoV-2 Infection Risk and Socioeconomic Disadvantage Among a Mayan-Latinx Population in Oakland, California. JAMA network open. 2021;4(5):e2110789.

35. Fan VS, Dominitz JA, Eastment MC, Locke ER, Green P, Berry K, et al. Risk Factors for Testing Positive for Severe Acute Respiratory Syndrome Coronavirus 2 in a National United States Healthcare System. Clinical infectious diseases : an official publication of the Infectious Diseases Society of America. 2021;73(9):e3085-e94.

36. Gershengorn HB, Patel S, Shukla B, Warde PR, Bhatia M, Parekh D, et al. Association of Race and Ethnicity with COVID-19 Test Positivity and Hospitalization Is Mediated by Socioeconomic Factors. Annals of the American Thoracic Society. 2021;18(8):1326-34.

37. Ioannou GN, Ferguson JM, O'Hare AM, Bohnert ASB, Backus LI, Boyko EJ, et al. Changes in the associations of race and rurality with SARS-CoV-2 infection, mortality, and case fatality in the United States from February 2020 to March 2021: A population-based cohort study. PLoS medicine. 2021;18(10):e1003807.

38. Khanna N, Klyushnenkova EN, Kaysin A. Association of COVID-19 With Race and Socioeconomic Factors in Family Medicine. Journal of the American Board of Family Medicine : JABFM. 2021;34(Suppl):S40-S7.

39. Mannheim J, Konda S, Logan LK. Racial, ethnic and socioeconomic disparities in SARS-CoV-2 infection amongst children. Paediatric and perinatal epidemiology. 2022;36(3):337-46.

40. Munoz-Price LS, Nattinger AB, Rivera F, Hanson R, Gmehlin CG, Perez A, et al. Racial Disparities in Incidence and Outcomes Among Patients With COVID-19. JAMA network open. 2020;3(9):e2021892.

41. Rozenfeld Y, Beam J, Maier H, Haggerson W, Boudreau K, Carlson J, et al. A model of disparities: risk factors associated with COVID-19 infection. International journal for equity in health. 2020;19(1):126.

42. Sakowicz A, Ayala AE, Ukeje CC, Witting CS, Grobman WA, Miller ES. Risk factors for severe acute respiratory syndrome coronavirus 2 infection in pregnant women. American journal of obstetrics & gynecology MFM. 2020;2(4):100198.

43. Vahidy FS, Nicolas JC, Meeks JR, Khan O, Pan A, Jones SL, et al. Racial and ethnic disparities in SARS-CoV-2 pandemic: analysis of a COVID-19 observational registry for a diverse US metropolitan population. BMJ open. 2020;10(8):e039849.
